# *Xenopus laevis* Kif18A is a highly processive kinesin required for meiotic spindle integrity

**DOI:** 10.1242/bio.023952

**Published:** 2017-02-22

**Authors:** Martin M. Möckel, Andreas Heim, Thomas Tischer, Thomas U. Mayer

**Affiliations:** Department of Molecular Genetics and Konstanz Research School Chemical Biology, University of Konstanz, Universitätsstraße 10, Konstanz 78457, Germany

**Keywords:** Kinesin-8, Kif18A, *Xenopus laevis*, Meiosis, Spindle structure

## Abstract

The assembly and functionality of the mitotic spindle depends on the coordinated activities of microtubule-associated motor proteins of the dynein and kinesin superfamily. Our current understanding of the function of motor proteins is significantly shaped by studies using *Xenopus laevis* egg extract as its open structure allows complex experimental manipulations hardly feasible in other model systems. Yet, the Kinesin-8 orthologue of human Kif18A has not been described in *Xenopus laevis* so far. Here, we report the cloning and characterization of *Xenopus laevis* (Xl) Kif18A. *Xenopus* Kif18A is expressed during oocyte maturation and its depletion from meiotic egg extract results in severe spindle defects. These defects can be rescued by wild-type Kif18A, but not Kif18A lacking motor activity or the C-terminus. Single-molecule microscopy assays revealed that Xl_Kif18A possesses high processivity, which depends on an additional C-terminal microtubule-binding site. Human tissue culture cells depleted of endogenous Kif18A display mitotic defects, which can be rescued by wild-type, but not tail-less Xl_Kif18A. Thus, Xl_Kif18A is the functional orthologue of human Kif18A whose activity is essential for the correct function of meiotic spindles in *Xenopus* oocytes.

## Abstract (precis)

**Summary:** The highly processive kinesin Kif18A, which is expressed during oocyte maturation in *Xenopus laevis*, is required for correct spindle formation in meiotic egg extracts and can functionally complement human Kif18A in tissue culture cells.

## INTRODUCTION

Kinesins are molecular motor proteins that convert the energy released by ATP hydrolysis into mechanical force (Vale and Milligan, 2000). Because of this characteristic feature, kinesins share a common motor domain of ∼350 amino acids (aa), which couples ATP hydrolysis to conformational changes resulting in altered affinities for microtubules (MTs). Based on phylogenetic analyses, the superfamily of kinesins has been classified into 14 different families (Miki et al., 2005). The Kinesin-8 family is unique in that it contains members that integrate two activities: movement towards the plus-ends of MTs and modulation of MT dynamics (Su et al., 2012). In mammalian cells, the Kinesin-8 member Kif18A accumulates at the plus-ends of kinetochore-MTs. Tissue-culture cells depleted of human (Hs) Kif18A display elongated spindles with hyper-stable MTs, chromosome congression defects and consequentially a spindle-assembly checkpoint (SAC)-dependent mitotic delay (Mayr et al., 2007; Stumpff et al., 2008). Efficient plus-end accumulation depends on both Kif18A's motor activity and an additional C-terminal MT binding site, which contributes to Kif18A's high processivity. Kif18A lacking the C-terminal MT binding site fails to rescue the mitotic defects in Kif18A-RNAi cells highlighting the importance of the plus-end accumulation of Kif18A for its mitotic function (Mayr et al., 2011; Stumpff et al., 2011; Weaver et al., 2011; Woodruff et al., 2010, 2012). Studies on the orthologues in *S. cerevisiae* (Kip3p) (DeZwaan et al., 1997; Straight et al., 1998; Su et al., 2011; Wargacki et al., 2010), *S. pombe* (Klp5/Klp6) (Garcia et al., 2002; West et al., 2002, 2001), and *D. melanogaster* (Klp67A) (Gandhi et al., 2004; Gatt et al., 2005; Goshima et al., 2005; Savoian et al., 2004; Savoian and Glover, 2010; Wang et al., 2010) suggest that the mitotic function of Kinesin-8 proteins is conserved. Yet, no information on *Xenopus laevis* Kinesin-8 Kif18A was available. Here, we clone and functionally characterize Xl_Kif18A. By combining *Xenopus* egg extract studies with *in-vitro* single molecule microscopy assays, we demonstrate that Xl_Kif18A possesses high processivity, which depends on an additional non-motor MT binding site at its C-terminus and which is important for its activity in regulating meiotic spindle function. We can furthermore show that the functional characteristics between human and *Xenopus* Kif18A seem to be conserved, as Xl_Kif18A can restore normal mitotic timing in human cultured cells depleted of endogenous Kif18A.

## RESULTS

### Xl*_*Kif18A is expressed during oocyte maturation

**Fig. 1.**
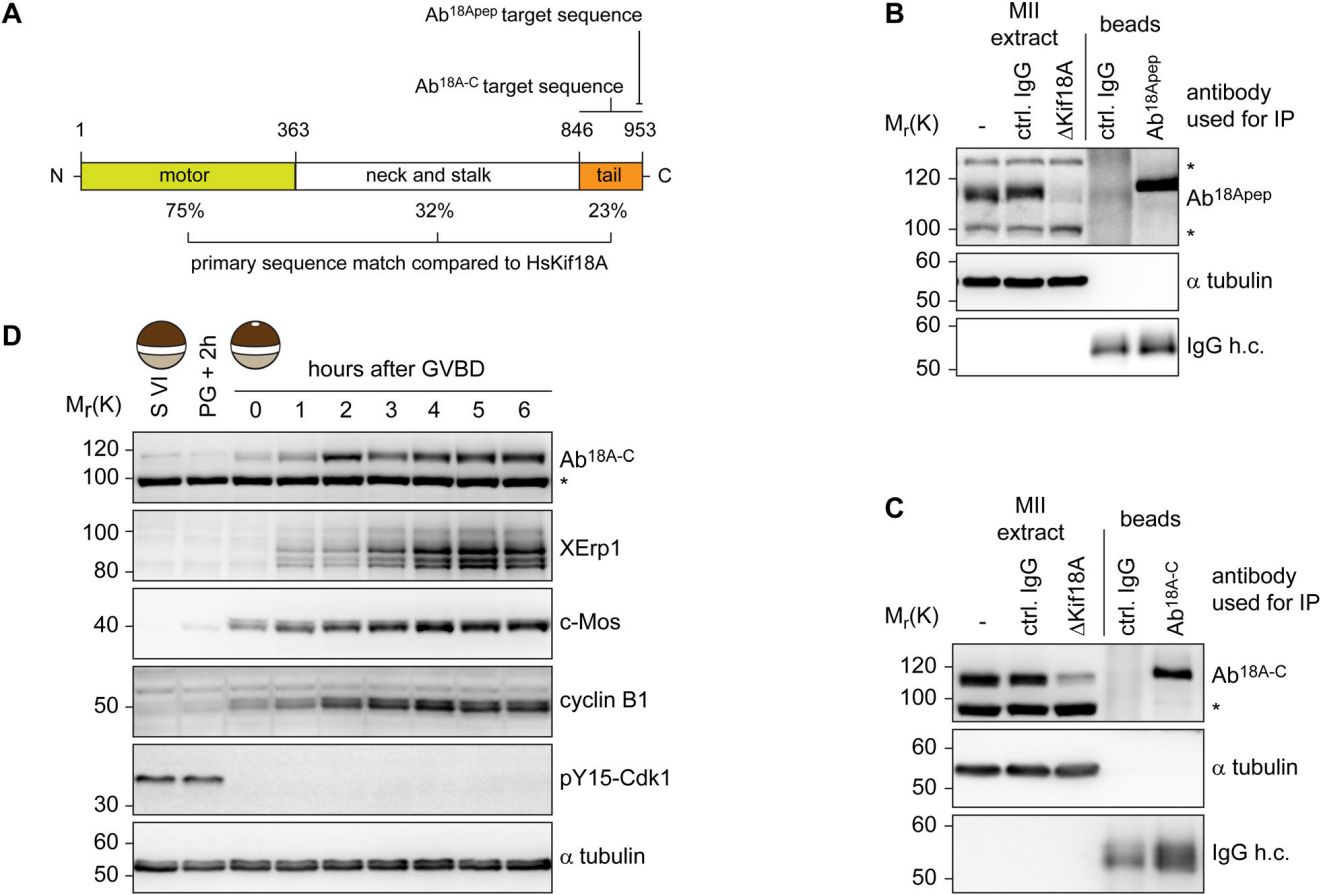
**Xl_Kif18A is expressed during female meiosis.** (A) Domain structure of Xl_Kif18A. (B,C) Immunoblot analyses of MII extract or bead samples after immunodepletion using Ab^18Apep^ (B) or Ab^18A-C^ (C). IgG antibodies were used as a control (Ctrl). (D) Immunoblot analyses of immature stage-VI (S VI) arrested oocytes before and at indicated time points after progesterone treatment using indicated antibodies.

To characterize *Xenopus laevis* Kif18A, we PCR amplified the open-reading-frame (ORF) of Kif18A using mRNA purified from mature *Xenopus* eggs and primers matching the annotated sequence of the start (exon three) and stop (exon 19) codon. The amplified ORF encoded a protein with 47% overall amino acid (aa) identity to Hs_Kif18A. Further sequence analyses identified an N-terminal motor domain with a Kinesin-8-characteristic, extended L2 loop and a C-terminal tail enriched for basic amino acids (Fig. S1). Next, we generated polyclonal antibodies against the last 11 C-terminal aa (943-953) of Xl_Kif18A (Fig. 1A). The purified antibody (Ab^18Apep^) recognized a band at the expected size of ∼110 kDa in extract from mature, metaphase-II arrested eggs (MII-extract, Fig. 1B). Immunodepletion using Ab^18Apep^ but not control IgG antibody resulted in reduced immunoreactivity in extract samples and enhanced signal intensity in the Ab^18Apep^ bead sample (Fig. 1B). Furthermore, an antibody raised against the first 103 amino acids of Xl_Kif18A (Ab^18AN^) detected a band at the same height of approximately 110 kDa in MII-extract, the signal of which was significantly reduced in Ab^18Apep^-immunodepleted extract and enhanced in the Ab^18Apep^ bead sample (Fig. S2A). These data suggest that Ab^18Apep^ specifically recognizes *Xenopus* Kif18A. Notably, we observed drastic variations in the abundance of Kif18A in MII-extracts prepared from different frogs. To understand the cause for this frog-to-frog variability, we prepared MII-extracts from eleven different frogs, analyzed these by immunoblotting (IB) and in parallel purified mRNA to analyze the Kif18A ORF. IB analyses (Fig. S2B) revealed a strong Kif18A signal in MII-extracts from eggs of frogs obtained from NASCO (#1 and #3-#6), while Kif18A was not detectable when in-house frogs were used (#2 and #7-#11). Intriguingly, frogs bred in-house differed from the annotated DNA sequence resulting in a Leu^950^ to Pro exchange within the antigen region, while Kif18A from NASCO frogs exactly matched the sequence (Fig. S2D,E). IB analyses of *in vitro* translated (IVT) C-terminal fragments of Kif18A (CT: aa 846-953) confirmed that the leucine to proline exchange interfered with the immunoreactivity of Ab^18Apep^ (Fig. S2C). Thus, due to a single nucleotide polymorphism, Kif18A was poorly detectable in egg extracts of in-house frogs and we therefore decided to raise another antibody against the C-terminus (aa 846-953) of Kif18A (Ab^18A-C^). Ab^18A-C^ was able to immunodeplete Kif18A from MII-extract (Fig. 1C) and detected Kif18A equally well in extracts prepared from eggs of in-house bred and NASCO frogs (Fig. S2F). Furthermore, Kif18A signal at the expected size of approximately 110 kDa was absent in Ab^18Apep^-immunodepleted egg extract (Fig. S2G) and increased in intensity after addition of *in vitro* translated Kif18A to egg extract (Fig. S2H) when probed with Ab^18A-C^. In summary, these data indicate that Ab^18A-C^ is a specific antibody for Xl_Kif18A detection in both in-house bred and NASCO frogs. Next, we analyzed the expression level of Kif18A during oocyte maturation. *Xenopus* immature oocytes (stage VI oocytes) are arrested at prophase-I until progesterone (PG) stimulation breaks this arrest and triggers the maturation of oocytes into fertilizable eggs arrested at metaphase of meiosis-II (Jessus and Ozon, 2004). Immunoblot analyses revealed that Kif18A was present at low levels in immature prophase-I oocytes, but accumulated as oocytes progressed through meiosis (Fig. 1D). Loss of inhibitory Cdk1 phosphorylation and accumulation of c-Mos, cyclin-B1 as well as XErp1 confirmed PG-induced meiotic maturation (Nishiyama et al., 2007; Schmidt et al., 2005).

### Xl_Kif18A is highly processive

**Fig. 2.**
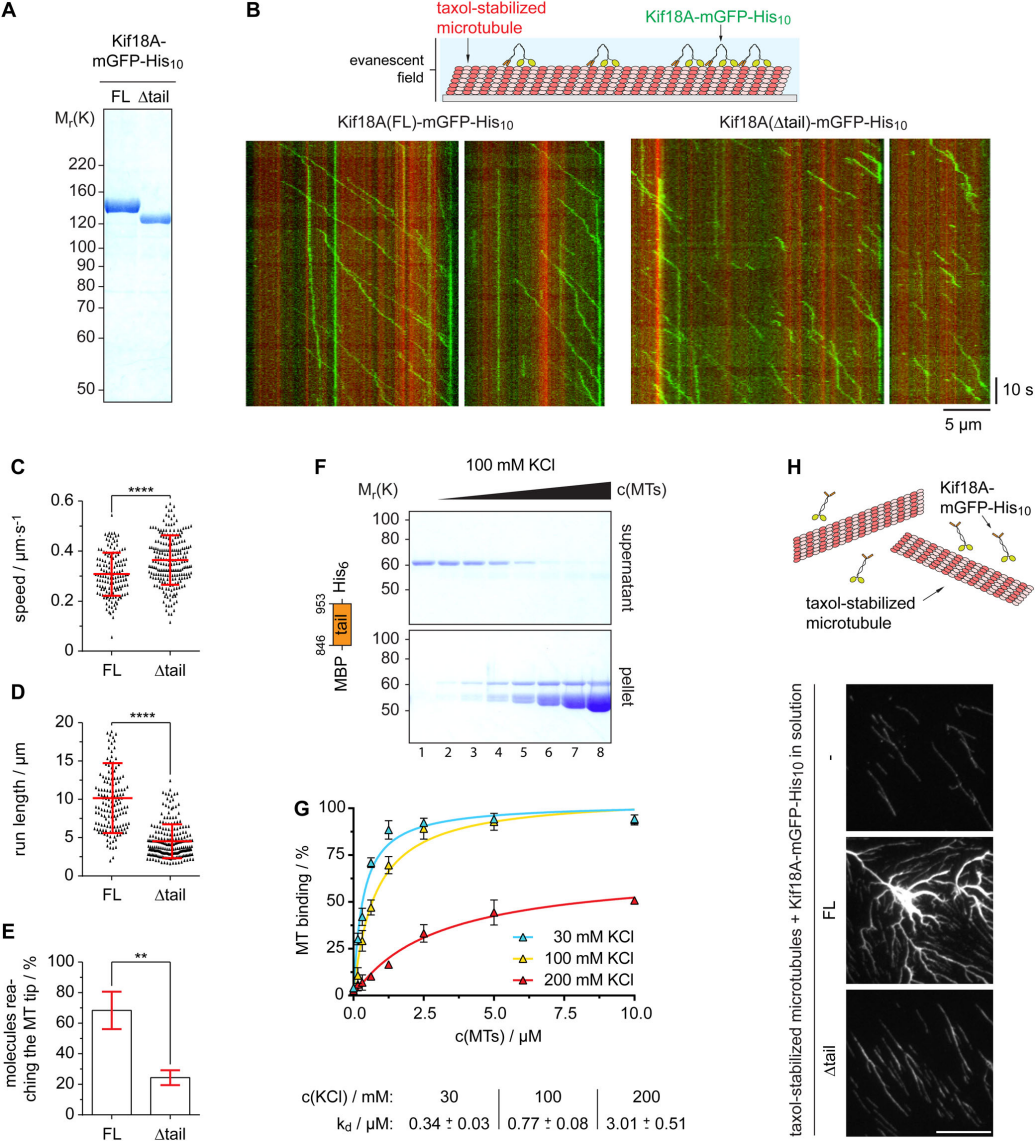
**Xl_Kif18A is a processive kinesin with an additional non-motor MT-binding site.** (A) SDS-PAGE analyses of recombinant full length (FL) and Δtail (aa 1-845) Kif18A-mGFP-His_10_. (B) Scheme of TIRF microscopy (top) and exemplary time (*y*-axis, scale bar: 10 s) versus space (*x*-axis; scale bar: 5 µm) plots (kymographs) of FL and Δtail Kif18A-mGFP-His_10_. (C) Speed and (D) run length of Kif18A-mGFP-His_10_ and (E) percentage of all molecules analyzed reaching the microtubule tip (mean±s.d. in red, unpaired *t*-test: ****P≤0.0001, **P≤0.01). (F) MT pelleting assay with MBP-Kif18A^CT^-His_6_ using varying concentrations of MTs (0-10 µM) and KCl (30-200 mM) analyzed by SDS-PAGE (from lane 1, 0 µM MTs, to lane 8, 10 µM MTs) and (G) quantified using ImageJ (mean±s.d., *n*=3 independent experiments, *K*_d_ values derived from one-site-specific binding fit in GraphPad Prism). (H) MT bundling assay using fluorescently labeled, taxol-stabilized MTs and nanomolar concentrations of Kif18A-mGFP-His_10_.

Next, we investigated the mechanochemical properties of Xl_Kif18A. To this end, we first purified full-length (FL) Kif18A fused at its C-terminus to monomeric green fluorescent protein (mGFP) and a His_10_-tag (Kif18A^FL^-mGFP-His_10_) from insect cells (Fig. 2A). A characteristic feature of human Kif18A and yeast Kip3p is their high processivity (Mayr et al., 2011; Stumpff et al., 2011; Su et al., 2011; Varga et al., 2006, 2009; Weaver et al., 2011). To test if *Xenopus* Kif18A shares this characteristic, we analyzed its processivity by TIRF-M (total-internal-reflection fluorescence microscopy) analyses (Fig. 2B). Analyses of time-space plots, so-called kymographs, revealed unidirectional movement of Kif18A^FL^-mGFP-His_10_ along taxol-stabilized MTs at a speed of 0.31±0.09 µm/s (Fig. 2B,C, Movie 1). The run length was 10.1±4.6 µm, confirming that *Xenopus* Kif18A shares the characteristic of high processivity (Fig. 2D). Consistently, a high percentage of Kif18A^FL^-mGFP-His_10_ molecules reached the tips of MTs that displayed an average length of 17±10 µm (Fig. 2E and Fig. S3A, respectively). The fluorescence intensity of the individual motile molecules appeared very similar (Fig. 2B) and the protein eluted as a single peak from a gel filtration column (Fig. S3B), indicating high homogeneity of the analyzed kinesin molecules. As shown previously, the high processivity of Kinesin-8 members depends on an additional, C-terminal MT binding site (Mayr et al., 2011; Stumpff et al., 2011; Su et al., 2013; Weaver et al., 2011). To test if the C-terminus of Xl_Kif18A directly binds to MTs, we performed MT pelleting assays. In the absence of MTs, the tail of Xl_Kif18A (aa 846-953, MBP-Kif18A^tail^-His_6_) remained in the SN fraction confirming the solubility of the fusion protein (Fig. 2F). With increasing concentrations of taxol-stabilized MTs, more MBP-Kif18A^tail^-His_6_ was found in the pellet fraction, while the tag control MBP-His_6_ remained in the SN fraction indicating that the interaction was mediated by Kif18A rather than the affinity tag (Fig. 2F, Fig. S3C,D). Intriguingly, increasing salt concentrations decreased the amount of MBP-Kif18A^tail^-His_6_ co-pelleting with MTs (Fig. 2G) indicating that the interaction is electrostatic. Notably, incubation of Kif18A^FL^-mGFP-His_10_ but not tail-less Kif18A (aa 1-845, Δtail) with taxol-stabilized MTs resulted in strong MT bundling (Fig. 2H) suggesting that, similar to yeast Kip3p (Su et al., 2013), the additional MT binding site enables Kif18A to crosslink MTs. Next, we tested if the C-terminal MT binding site contributes to Kif18A's high processivity. TIRF-M analyses of Kif18A^Δtail^-mGFP-His_10_ (Fig. 2A and Fig. S3B) revealed that the run length was significantly decreased compared to the FL protein, while the velocity was slightly increased (Fig. 2B-D and Movie 2). In accordance with the reduced processivity, the percentage of Kif18A^Δtail^-mGFP-His_10_ molecules reaching the MT tips was strongly reduced (Fig. 2E; average MT length: 15±9 µm Fig. S3A). In summary, the C-terminal tail of Kif18A possesses an additional MT binding site that interacts with MTs in an electrostatic manner and contributes to Kif18A's high processivity, enabling its accumulation at the tips of MTs.

### Xl_Kif18A is important for its meiotic spindle function

**Fig. 3.**
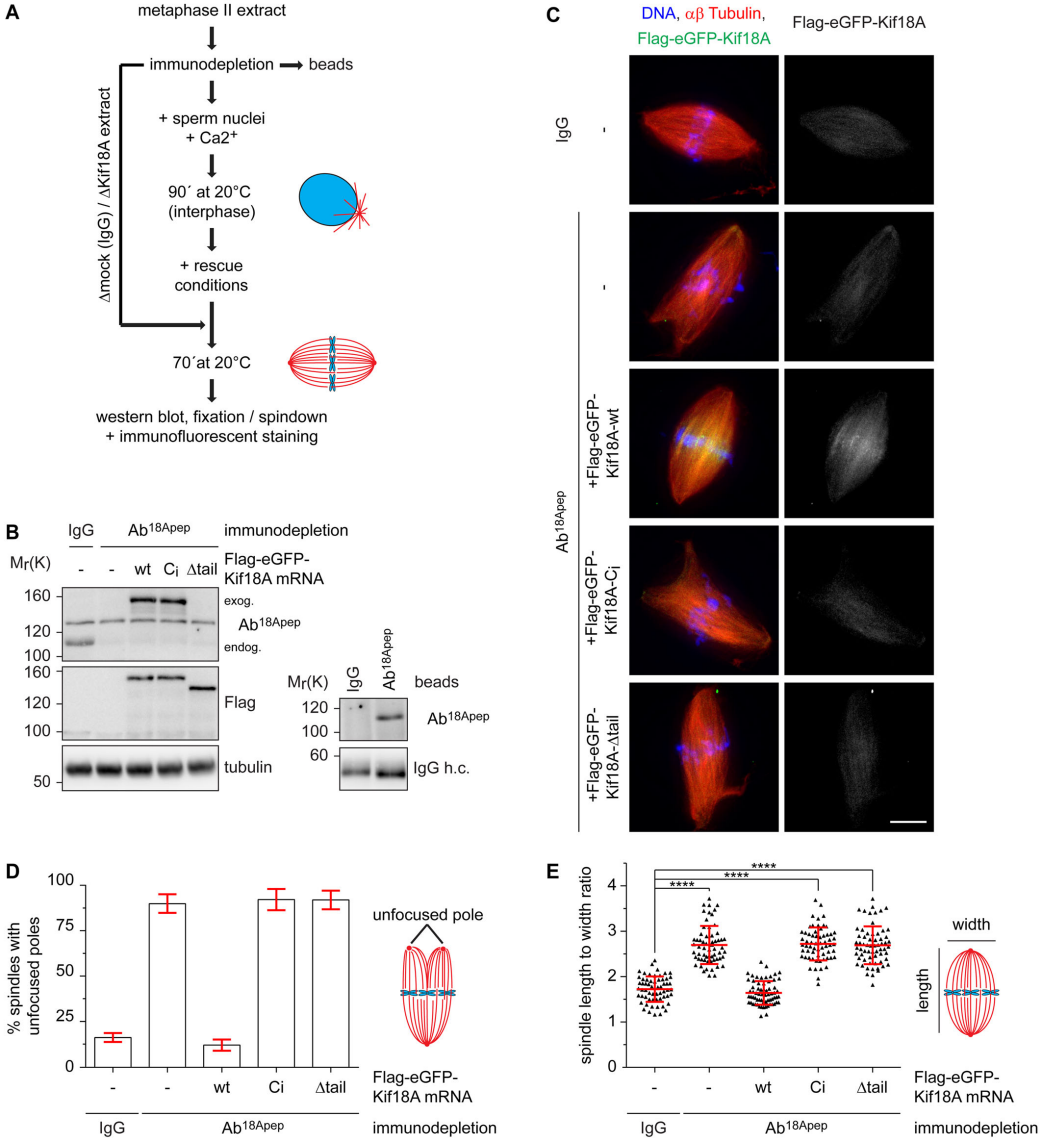
**Xl_Kif18A is important for meiotic spindle structure.** (A) Scheme of the depletion /add-back experiments. (B) Immunoblot of control (IgG)- or Kif18A (Ab^18Apep^)-depleted extracts supplemented with mRNA encoding wt, C_i_ or Δtail Flag-eGFP-Xl_Kif18A. Right panel shows immunoblot of IgG or Ab^18Apep^ beads. (C) Representative fluorescence images of spindles obtained as described in A. DNA, αβ-tubulin, and Flag-eGFP-Xl_Kif18A are shown in blue, red and green, respectively. Scale bar: 10 µm. (D) Quantification of spindle length to width ratio. (E) Quantification of spindles with multiple/unfocused poles (more than 60 spindles analyzed per condition, mean±s.d., unpaired *t*-test: ****P≤0.0001).

Kif18A is expressed during oocyte maturation (Fig. 1D) indicating that it might be required for meiotic spindle function. Injection of morpholino oligonucleotides targeting Xl_Kif18A into immature oocytes followed by PG treatment did not result in significantly reduced Kif18A levels (data not shown). Therefore, we used *Xenopus* egg extract to investigate if Xl_Kif18A is important for meiotic spindle function. In brief, Kif18A- or control-depleted MII extract supplemented with sperm nuclei as source for centrosomes and chromatin was released into interphase by calcium treatment and subsequently induced to re-enter M-phase by the addition of Kif18A- or control-depleted MII extract (Fig. 3A). Using Ab^18Apep^ for two rounds of depletion, Kif18A levels were greatly reduced from egg extract prepared from Nasco frogs (Fig. 3B). Compared to Ctrl extract, Kif18A-depleted extract displayed more frequently slightly longer and thinner spindles with asymmetric shapes and unfocused spindle poles (Fig. 3C-E). Unfortunately, we were not able to detect endogenous Kif18A on extract-derived spindles with any of the antibodies used in this study. To quantify the spindle phenotype, we determined the length to width ratio of assembled spindles and indeed this value was significantly increased in Kif18A-depleted extracts compared to control extracts (Fig. 3E). Supplementing interphase extract with mRNA encoding wild-type (wt) Flag-enhanced (e)GFP-Kif18A^FL^ (Fig. 3B), rescued the spindle defects (Fig. 3C-E) confirming that the phenotypes were specific for Kif18A depletion. Consistent with the idea that Kif18A requires both its motor activity and non-motor MT binding site for plus-end localization, wt Flag-eGFP-Kif18A^FL^ but neither catalytically inactive (C_i_) nor tail-less (Δtail) Kif18A accumulated in the proximity of chromatin in extracts depleted of endogenous Kif18A (Fig. 3C). And consequentially, Kif18A^Ci^ and Kif18A^Δtail^ failed to rescue the spindle defects (Fig. 3C-E). We conclude that the motor activity and high processivity of Xl_Kif18A is important for its function in regulating spindle morphology in meiotic *Xenopus* egg extract.

### Xl_Kif18A can functionally complement human Kif18A

**Fig. 4.**
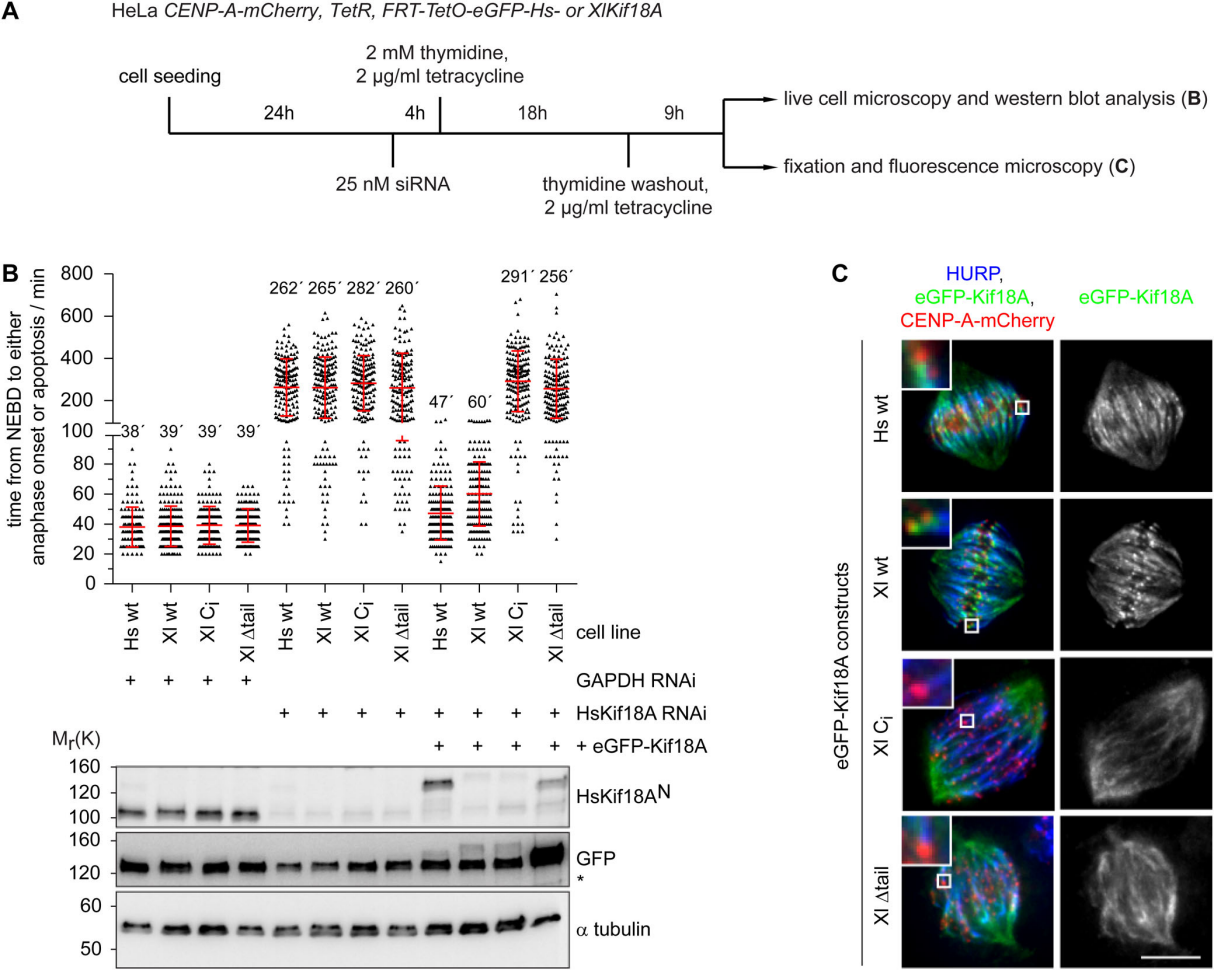
**Xl_Kif18A can complement the function of human Kif18A.** (A) Scheme of the RNAi/rescue experiments using HeLa cells expressing constitutively CENP-A-mCherry and inducibly siRNA resistant human or *Xenopus* eGFP-Kif18A. (B) Quantification of mitotic timing (timing from NEBD to either commitment to anaphase onset or apoptosis; top) in cell lines treated as described in A using fluorescent time-lapse imaging (time resolution 5 min, more than 200 cells per condition, mean±s.d. in red, *n*=3 independent experiments) and immunoblot analyses (bottom) of respective cells. Unspecific band is marked with an asterisk. (C) Representative fluorescence images of spindles in mitotic cells expressing eGFP-Kif18A constructs as indicated. HURP, CENP-A-mCherry, and eGFP-Kif18A are shown in blue, red and green, respectively. Insets show magnified view of area marked with white box. Scale bar: 10 µm.

The observed meiotic spindle phenotype and mechanochemical properties suggest that Xl_Kif18A is the functional orthologue of human Kif18A. To test this idea directly, we performed RNA-interference (RNAi) rescue studies in HeLa cells (Fig. 4A). HeLa-cells transfected with short-interfering RNA (siRNA) targeting the Kif18A ORF displayed greatly reduced levels of Kif18A (Fig. 4B). Live cell analyses using CENP-A-mCherry to visualize centromeres revealed that Kif18A-RNAi cells spent significantly longer time in mitosis [time from nuclear envelope breakdown (NEBD) to either anaphase onset or apoptosis after an elongated mitotic arrest] than control depleted cells (262±135 min versus 38±13 min, Fig. 4B). As expected, expression of human, siRNA-resistant eGFP-Hs_Kif18A rescued mitotic timing (47±18 min, Fig. 4B). Intriguingly, expression of *Xenopus* wt eGFP-Xl_Kif18A restored mitotic timing to almost control levels (60±21 min, Fig. 4B). To analyze the localization of Xl_Kif18A, cells were chemically fixed and stained for HURP, a mitotic spindle protein that localizes to kinetochore-attached microtubule fibers (k-fibers) in the vicinity of chromosomes (Koffa et al., 2006; Silljé et al., 2006). eGFP-Xl_Kif18A^wt^ localized to k-fibers in a comet-like fashion, comparable to its human orthologue (Fig. 4C). Under these conditions, spindles displayed normal morphologies with correctly aligned chromosomes (Fig. 4C). Consistent with the results obtained in *Xenopus* egg extract, the ability of *Xenopus* Kif18A to complement the function of its human orthologue strictly depended on its catalytic activity and non-motor MT binding site. Both C_i_ and Δtail mutants failed to concentrate at the plus tips of k-fibers (Fig. 4C) and to restore mitotic timing (291±144 min and 256±140 min, respectively, Fig. 4B), which was accompanied by aberrant spindle structures and misaligned chromosomes as shown by immunofluorescence analyses (Fig. 4C).

## DISCUSSION

Members of the Kinesin-8 family are important for the function of the spindle apparatus during M-phase from yeast to humans. In this study we identified the *Xenopus* orthologue of Kif18A, one of the best-studied Kinesin-8 members, that regulates k-fiber length and hence chromosome alignment during mitosis in human cultured cells (Mayr et al., 2007; Stumpff et al., 2008).

While previous studies in primary cells underline the importance of Kif18A function mainly during male meiotic divisions (Gandhi et al., 2004; Liu et al., 2010; Savoian et al., 2004), it also seems to be required during germ cell divisions in female mice (Czechanski et al., 2015). Given the upregulation of Kif18A expression during female meiosis (Fig. 1D) and the disruption of spindle integrity in female meiotic egg extracts in the absence of Kif18A (Fig. 3), our data suggest an important role of Kif18A during female meiotic divisions also in the African clawed frog *Xenopus laevis*. Unfortunately, attempts to downregulate Kif18A levels in intact oocytes using morpholino antisense oligos have not been successful so far (not shown). Inactivation of Kif18A function in oocytes and subsequent phenotypic analysis thus remain important tasks for future research.

Previous studies suggested that highly processive MT-plus end directed Kif18A molecules accumulate at the plus ends of k-fibers, where they dampen microtubule dynamics or even induce catastrophe, resulting in suppression of chromosome oscillations prior to anaphase onset in human cells (Du et al., 2010; Stumpff et al., 2011). Our data underline conserved mechanochemical properties: *Xenopus* Kif18A is highly processive (Fig. 2B,D), which can at least partially be attributed to a second, non-motor microtubule binding site in its C-terminal tail region (Fig. 2B,D,F), and presumably accumulates at microtubule plus tips of spindles generated in meiotic egg extract (Fig. 3C). It is therefore surprising that Kif18A depletion from meiotic egg extract leads to unfocused spindle poles (Fig. 3C,D). The increase in splayed spindle poles in the absence of Kif18A might be explained by the ability of the kinesin to bundle microtubules (Fig. 2H) and thereby regulate spindle morphology in meiotic *Xenopus* egg extract. Possibly, the unique situation in *Xenopus* egg extract where spindles are not attached to the cell cortex might also contribute to the spindle phenotype in the absence of Xl_Kif18A.

High mechanistical and functional conservation between human and *Xenopus* Kif18A is further underlined by the finding that the *Xenopus* version is able to complement the function of its human orthologue in HeLa cells (Fig. 4). Like human Kif18A, the ability of Xl_Kif18A to fulfill its spindle function depends on both the motor activity and the non-motor MT binding site of the kinesin (Fig. 4B,C), indicating that high processivity is a key feature of Xl_Kif18A′s mode of action. Interestingly, compared to wild-type Kif18A, the truncated construct lacking the C-terminal non-motor MT binding site is much more abundant in human cells (Fig. 4B). Elements required for the regulation of Kif18A stability therefore likely lie in these last 107 aa of the kinesin, as shown for human Kif18A (Sedgwick et al., 2013).

We were not able to show a direct effect of Xl_Kif18A on MT dynamics *in vitro*, as described earlier for orthologues in yeast and human (Mayr et al., 2007; Stumpff et al., 2011; Varga et al., 2006, 2009). However, depletion of Kif18A from meiotic extracts results in slightly elongated and/or thinner spindles (Fig. 3C,E) and the kinesin can revert the hyper-elongated mitotic spindle phenotype in human cells (Fig. 4C), suggesting that Xl_Kif18A shares with human Kinesin-8 the ability to regulate MT plus-end dynamics. Further studies are required to dissect the detailed molecular mechanisms by which Kif18A controls the length of MTs within the meiotic spindle of *Xenopus laevis*.

## MATERIALS AND METHODS

### Plasmids and antibodies

Full-length Xl_Kif18A was cloned from egg cDNA using primers 5′-attaggccggcccatggaagcctcccaagaggacgtc-3′ and 5′-taatggcgcgccctcagggatctttaagggctgc-3′. Rabbit polyclonal antibodies were generated against a C-terminal peptide: N-CGGRKKAALKDP-C (Ab^18Apep^), a protein fragment containing an N-terminal Serine: aa 845-953 (Ab^18A-C^) and a fragment from aa 1-104 tagged with an N-terminal His6-SMT3 (Ab^18AN^). Other antibodies used were: human Kif18A^N^ (Mayr et al., 2007; 1 µg/ml), anti-GFP (Covance, MMS-118P, 1:1000); anti-cyclin B2 (Abcam, ab18250; 1:1000), anti-XErp1 (Schmidt et al., 2005; 1 µg/ml); anti- c-Mos (Santa Cruz, C237; 1:250), anti pY15-Cdk1 (Cell Signaling, 9111, 1:1000); anti-Flag (Sigma, F1804, 1:1000); anti α-tubulin (Sigma, F2168, 1:2000), anti HURP (Abcam, ab70744, 1:500), anti-mouse IgG-HRP and anti-rabbit IgG-HRP (Dianova, 115-035-062 and 111-035-003, both 1:3000).

### Protein purification

His6-TEVsite-tagged and MBP-His6-tagged Kif18A fragments were expressed in *E.coli* BL21-RIL, mGFP-His10-tagged Kif18A in SF9 insect cells using the Bac-to-Bac Baculovirus system (Invitrogen). Proteins were purified using Ni-IDA Resin (Macherey-Nagel). Gel filtration was performed using an Äkta-Purifier FPLC and Superdex-200 10/300 and Superose-6 10/300 as described in Möckel et al. (2016).

### Microtubule assays

MTs were prepared as described previously (Möckel et al., 2016). Assays were performed in TIRF assay buffer with varying KCl concentrations. Kif18A (1 µM) and MTs (0 to 10 µM) were incubated (10 min, 28°C), tubes were spun for 5 min at 20,000 ***g*** and SDS-PA gels loaded with SN and P fractions were stained with CBB. Kif18A band intensities for supernatant (iSN) and pellet (iP) fractions were measured using ImageJ. The percentage of MT-bound Kif18A (%bound) was calculated by: iP×(iSN+iP)^−1^×100. Three independent experiments were analyzed for all conditions. *K*_d_ values were derived from a one-site-specific binding fit in GraphPad Prism.

### TIRF assays

Fluorescent kinesin molecules were imaged on surface-linked MTs in a TIRF field as described earlier (Möckel et al., 2016). Motility buffer contained 75 mM KCl and BRB20. Kif18A-mGFP-His_10_ concentration was 1 nM.

### Microtubule bundling assays

50 nM Xl_Kif18A-mGFP-His_10_ was incubated with Atto595-labeled microtubules (tubulin dimer concentration of 250 nM) in TIRF assay buffer for 10 min, 28°C. Fixation buffer (3.7% formaldehyde, 40% glycerol in BRB80) was added and the reaction was spun on coverslips through a cushion (60% glycerol in BRB80). Coverslips were washed (PBS, 0.1% Triton X-100) prior to mounting in Mowiol.

### *Xenopus* egg extract spindles

Metaphase spindles were generated as previously described (Maresca and Heald, 2006). Kif18A was immunodepleted from CSF extract using Ab^18Apep^ coupled to Dyna Protein-G beads (Invitrogen) in two subsequent rounds, using 20 µg antibody. mRNAs coding for Flag_3_-eGFP-Kif18A variants were added (3-5 ng/µl final).

### Cell assays

Stable HeLa (ATCC) cell lines inducibly expressing siRNA resistant Kif18A variants were generated using the Flp-In/T-REx system (Invitrogen). Transfection of siRNA targeting human Kif18A and time-lapse microscopy was performed as previously described (Häfner et al., 2014). High-resolution analysis of mitotic spindles was performed as previously described in Mayr et al. (2007).
